# Molecular epidemiological study of hepatitis B virus in blood donors from five Chinese blood centers

**DOI:** 10.1007/s00705-012-1331-x

**Published:** 2012-06-06

**Authors:** Yu Liu, Jingxing Wang, Yi Huang, Tonghan Yang, Xiaoming Guo, Julin Li, Guoxin Wen, Zhongqiao Yun, Peibin Zeng, Miao He, Min Xu, Gui Liu, Ling Ke, David Wright, Jing Liu, Kenrad Nelson, Hua Shan

**Affiliations:** 1 Institute of Blood Transfusion, Chinese Academy of Medical Sciences, Hua Cai Road 26 Hao, Dong San Huan Road Er Duan, Chengdu, 610052 Sichuang China; 2 Yunnan Kunming Blood Center, Kunming, Yunnan China; 3 Mianyang Blood Center, Mianyang, Sichuan China; 4 Guangxi Blood Center, Liuzhou, Guangxi China; 5 Urumqi City Blood Center, Urumqi, Xinjiang China; 6 Luoyang Blood Center, Luoyang, Henan China; 7 Westat, Rockville, MD USA; 8 Johns Hopkins University, Baltimore, MD USA

## Abstract

Although the genetic variability of hepatitis B virus (HBV) in HBV-infected patients has been extensively studied, reports on genotypes, subtypes and mutations in the S region of HBV strains from Chinese blood donors are limited. In this study, 245 blood samples from HBsAg-positive blood donors were collected from five geographically diverse blood centers in China. The S region of HBV was amplified, and the HBV genotype and subtype were determined. The amino acid sequences of the S region were aligned, and mutations related to the failure of immunization and HBsAg detection were determined. Of the 245 samples, 228 (93 %) were genotyped successfully. We found that genotypes B, C, D and A accounted for 58.8 %, 21.9 %, 6.6 % and 3.95 % of the isolates, respectively. The distribution of HBV antigen subtypes was as follows: adw (67.6 %), adr (23.3 %) and ayw (8.7 %). Mutations were present in 39 (17.1 %) of 228 samples in the major hydrophilic region (MHR) of the S region. This study demonstrated that HBV genotype/subtype B/adw was the most frequent strain circulating in HBV-infected Chinese blood donors, followed by C/adr. The occurrence of MHR mutants in HBV-infected blood donors and the potential failure to detect some of them in collected units poses a threat to transfusion safety.

## Introduction

Hepatitis B virus (HBV) is an aetiological agent of acute and chronic liver disease, including fatal fulminant hepatitis, cirrhosis and hepatocellular carcinoma, which is one of the most common human cancers and causes of death worldwide [1]. It has been estimated that more than 2 billion of the global population have been infected with HBV. Of these, approximately 360 million people are chronically infected, and an estimated 500,000 to 700,000 people die from complications of HBV infection each year worldwide [2]. HBV infection is highly endemic in China, with 5.84 % prevalence of HBV surface antigen (HBsAg) in the population of 1-59 years of age in 2007 [3].

HBV demonstrates remarkable genetic variability. Historically, using hepatitis B surface antigen (HBsAg) subtyping techniques based on specific antibodies, the genetic variability of the HBV envelope allowed classification into ten serologic subtypes, designated ayw1, ayw2, ayw3, ayw4, ayr, adw2, adw3, adw4, adrq+, and adrq– [4]. More recently, HBV has been classified into eight genotypes (A to H, in the order of discovery) with each genotype differing from the others by 8 % nucleotide divergence in the complete genome and 4 % in the sequence of the S gene [4]. Currently, HBV subtypes can be deduced from amino acid sequences at positions 122, 127, 134, 159, 160, 177 and 178 [5]. HBV genotypes have a distinct geographical distribution [6, 7]. For genotype A, subgenotype A2 is found mainly in northwestern Europe, North America and in Australians of European origin, while the more prevalent A1 is found in East and South Africa and along the coast of the Indian Ocean; genotypes B and C are prevalent in Southeast Asia, China and Japan; genotype D is spread worldwide, but it is predominant in the Mediterranean region and the Middle East; genotype E is almost entirely restricted to Africa; genotype F is found in Central and South America; genotype G has been reported in France and North America; and genotype H predominates in Central America. Recently, a ninth genotype (I) was tentatively proposed for HBV strains detected in Laos and a tenth genotype (J) was proposed for a HBV strain detected in a Japanese HCC patient [8, 9]. In China, genotypes C and B are predominant in patients with chronic liver disease, followed by genotypes D, E and A [10].

Besides genotype diversity, HBV S-gene mutations have been reported to affect nearly all amino acid positions of the major immunogenic region, the “a” determinant, which spans residues 124–147 of HBsAg. These mutations can lead to a false negative result when testing for HBsAg in blood donations and thus impact blood safety, especially in countries, including China, where screening of antibody to hepatitis B core antigen (anti-HBc) or HBV DNA is not routinely done [10].

Because the prevalence and sequelae from chronic HBV infections are very high in China, the Chinese government has taken effective measures to establish a universal infant immunization program to prevent HBV infections since 1992. Since then, hepatitis B vaccine coverage has reportedly increased from 30.0 % for newborns in 1992 to 93.4 % in 2005 [11]. However, nearly 20 years after the vaccination policy was put into effect in China, no comprehensive study of the HBV genotype prevalence or S-gene mutations in Chinese blood donors has been conducted. Little is known about mutations in the MHR region of HBV from blood donors. In this study, we analyzed the HBV genotype/subtype and mutations in the S region from voluntary blood donors who attempted to donate in five geographically diverse Chinese blood centers that participated in the Retrovirus Epidemiology Donor Study-II (REDS-II) International-China Program. The REDS-II International Program was developed to improve international blood safety by studying infectious and non-infectious risks of blood transfusion and mechanisms to improve the successful recruitment of low-risk volunteer blood donors. All blood donors participating in the REDS-II International China studies were provided with information on the objectives and nature of the studies, and they gave their written consent before enrolling in the studies. All REDS-II study protocols were reviewed and approved by the IRB at Johns Hopkins University and Chinese Academy of Medical Sciences prior to the implementation of the protocols.

## Materials and methods

### Samples

Five blood centers in China including the Yunnan Kunming Blood Center (Kunming, Yunnan), Urumqi Blood Center (Urumqi, Xinjiang), Luoyang Blood Center (Luoyang, Henan), Mianyang Blood Center (Mianyang, Sichuan), and Liuzhou Blood Center (Liuzhou, Guangxi) participated in the NHLBI-sponsored REDS-II International-China Program. Blood samples from donors who attempted to donate blood at these five blood centers between 2008 and 2010 and consented to participate in the REDS-II program were tested twice by ELISA for HBsAg using ELISA kits made by two different manufactures (The kit produced by Shanghai Kehua was used by the Kunming, Urumqi and Luoyang blood centers for the first-round ELISA, and the kit from Xinchuang was used by the other two blood centers for the first-round detection. For the second-round ELISA, the kit produced by Jinhao was used by the Urumqi blood center, the kit from Xinchuang was used by the Kunming and Luoyang blood centers, and the kit from Abbott was used by the other two blood centers. Samples that were reactive with either one or both of these screening ELISA kits were tested again using a third ELISA kit (Monolisa HBsAg ULTRA Assay; Bio-Rad, France) as a surrogate measure for confirmation of HBV infection. Samples confirmed by the third ELISA testing were included in this study. The reactive whole-blood samples were collected in two ethylenediaminetetraacetate (EDTA)-K2 (with separator gel) vacuum tubes (Greiner, Kremsmünster, Austria) at the blood-collection sites. Plasma was separated from the RBCs by centrifugation at 4 °C. Samples were then frozen and shipped on dry ice to the Institute of Blood Transfusion (IBT), Chinese Academy of Medical Sciences. To avoid repeat freezing and thawing, all of the plasma transported to IBT was divided into 1.5-mL tubes and stored at −70 °C.

The basic demographic information about the donors was obtained from the REDS-II China database [12]. From 2008 to 2010, a total of 3,240 HBsAg-reactive samples were collected, and up to 10 samples were selected randomly from each blood center every half year. Each month, the first two HBsAg-reactive samples were selected at each center. If more than ten samples were selected in a half year for the first-round selection, only the first ten samples were included in the study. If fewer than ten samples were selected within a half year for the first-round selection, additional samples were selected from the months when more than two reactive samples were collected. Because either no samples or fewer than ten HBsAg-reactive samples were collected in some half years from some centers, only 245 HBsAg-positive samples were selected for this study.

### Viral DNA extraction and amplification of the S region

HBV DNA was extracted from 200 μl plasma using a QIAamp® DNA Blood Mini Kit (QIAGEN, Hilden, Germany) according to the manufacturer’s instructions, and 50 μl of eluted DNA was stored at −70 °C until use. The HBV S region was amplified by nested PCR using a thermal cycler (Veriti, Applied Biosystems) with BS1 (nt 203-221, 5′-GCGGGGTTTTTCTTGTTGA-3′) as the sense primer and BS2 (nt 788-769, 5′-GGGACTCAAGATGTTGTACAG-3′), and BS3 (nt 712-731, 5′-AAGCCCTACGAACCACTGAA-3′) as the antisense primers for the first-round and second-round PCR, respectively. The first-round PCR was performed with Taq DNA polymerase (Tiangen) in a total volume of 40 μL, with the following reaction variables: predenaturation at 95 °C for 5 minutes, followed by 35 cycles of 15 seconds of denaturation (95 °C), 30 seconds of annealing (53 °C), and 30 seconds of extension (72 °C), with a final extension at 72 °C for 5 minutes. The cycling conditions of the second-round PCR were the same as the first-round PCR but using 2 μL of the first-round PCR product as template. PCR cycling was performed on a thermal cycler (Veriti, Applied Biosystems). To avoid false positive results, comprehensive procedures were followed to prevent sample cross-contamination, and test results were accepted as valid only when obtained in duplicate.

### DNA sequencing, phylogenetic analysis and multiplex PCR

The PCR products were analyzed by electrophoresis in 1.5 % agarose gels and purified using a commercially available kit (NucleoSpin Extract II kit, Macherey-Nagel GmbH & Co. KG, Düren, Germany) according to the manufacturer’s instructions. Purified products were used as templates in cycle sequencing reactions. Nucleotide sequences were determined from both strands using primers BS1 and BS3, and the resulting sequences were read directly with a genetic analyzer (ABI 3730, Applied Biosystems). For the single sequence with no heterogeneous sites, the HBV genotypes were determined by phylogenetic analysis with a panel of reference sequences from genotypes A to H retrieved from GenBank. The GenBank accession numbers for the reference strains were AB076678, AF536524, AJ012207, and AB194951 for genotype A; AB073851, AB106885, AB010290, AB205119, AB073834, AY293309, AF121245, AB033554, AB031267, AB219426, and AB219427 for genotype B; AB074047, AF223961, AB031262, AB014374, AB014360, AB026814, X75656, AB105172, GQ377635, Y18855, AB241110, AB241109, and AF241410 for genotype C; AY161157, AF151735, AB078033, AB090269, AJ132335, AY902776, AB048701, AB033559, DQ315779, and DQ315780 for genotype D; AB091255 and AB194948 for genotype E; AB036909 and AY090458 for genotype F; AB064311 and 064313 for genotype G; and AY090457 for genotype H. Nucleotide sequences were multiply aligned using the CLUSTAL_X (version 1.83) program [13]. The alignments were then used to construct phylogenetic trees for each subalignment using the neighbor-joining method implemented by the MEGA program [14]. The statistical validity of the neighbor-joining trees was assessed by bootstrap re-sampling with 1000 replicates. If the amplified sequence had heterogeneous sites identified by sequencing both strands, multiplex PCR was used for genotyping as reported previously [15].

### Analysis of subtypes and mutations in the S region

The distribution of the HBV subtypes was deduced from the amino acid sequences at positions 122, 127, 134, 159, 160, 177 and 178 [16]. The presence of amino acid mutations in the antigenic loop was analyzed from amino acid 103 to 173 of HBsAg and compared to a consensus sequence from the same genotype. Mutations related to genetic polymorphisms were noted, but the focus was placed on mutations reported to be associated with vaccine escape, diagnostic failure, treatment failure with HBsAg immune globulins, or resistance to antiviral therapy [17, 18].

### Statistical analysis

Data were expressed as mean ± SD and percentages, as appropriate, with 95 % confidence intervals (95 % CI). Comparisons between groups were analyzed by chi-square test or Fisher’s exact test for categorical variables and by Student’s t-test for quantitative variables. P-values below 0.05 were considered significant. All statistical analysis was performed using SPSS software for Windows 10.0 (SPSS, Chicago, IL).

## Results

A total of 228 (93.1 %) of the 245 randomly selected HBsAg-positive samples were successfully genotyped. Of these, 146 samples were sequenced directly, and the mean donor age was 28.3 ± 8.8 years. Eighty-two samples were genotyped by multiplex PCR, and the mean donor age was 32.0 ± 10.2 years. The donors from the second group were significantly older than those from the first group (p = 0.004). The sequences from the second group samples were found by direct sequencing to have heterozygous loci, which accounted for 36.0 % of the successfully genotyped samples. Overall, 24.4 % of the samples with heterozygous loci were found to contain mixtures of genotypes (n = 20), while the rest represented mixtures of variants of a single genotype (n = 62). Of the donors with genotype mixtures, genotype B and C mixtures represented 75.0 % of the total (n = 15), followed by genotype A and B mixtures (n = 3) and genotype B and D mixtures (n = 2).

Of the 228 blood donors genotyped successfully, we found nine donors infected with genotype A (3.95 %, 95 % CI: 1.4 %-6.4 %), 134 with HBV genotype B (58.8 %, 95 % CI: 52.4 %-65.2 %), 50 with genotype C (21.9 %, 95 % CI: 16.6 %-27.3 %), 15 with genotype D (6.6 %, 95 % CI: 3.4 %-9.8 %), 20 (8.8 %, 95 % CI: 5.1 %-12.4 %) with two genotypes (genotype mixture), and no subjects with genotypes E to H.

### Geographic distribution of HBV genotypes and subtypes

**Fig. 1:**
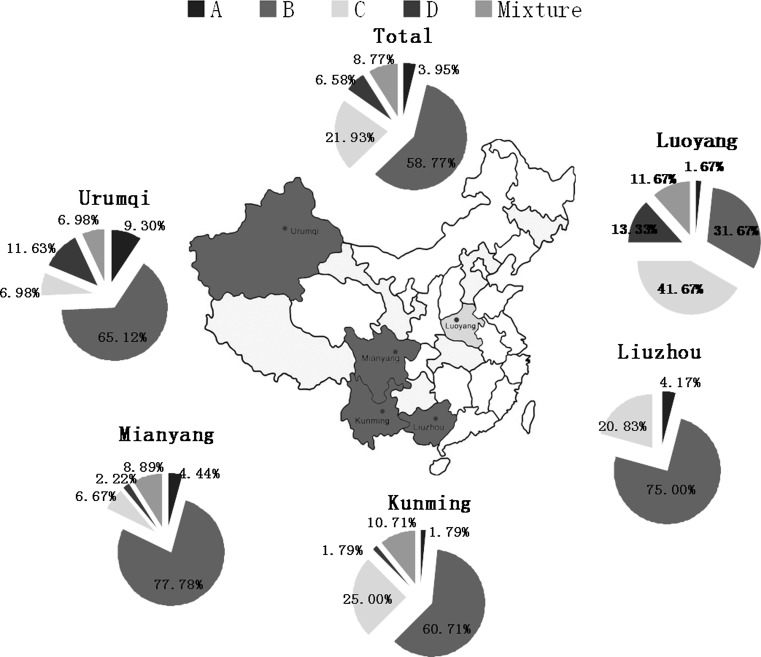
Distribution of HBV genotypes in five blood centers. In this figure, **A**, **B**, **C** and **D** represent genotypes A, B, C and D, respectively, and “mixture” refers to a mixture of at least two different genotypes. Genotype B was the most common genotype in all blood centers except for the Luoyang Blood Center, where the prevalence of genotype C was higher than that of genotype B

The geographic distribution of HBV genotypes varies as shown in Fig. 1 (p < 0.0001). HBV genotypes B and C were predominant in four of the five blood centers (Fig. 1). Genotype B was the most common genotype in all blood centers except for the Luoyang Blood Center, where the prevalence of genotype C was higher than that of genotype B. In Urumqi, however, a higher proportion of genotype D than C (11.63 % vs. 6.98 %) was found. The proportion of genotype D was significantly higher in Urumqi Blood Center (11.63 %) and Luoyang Blood Center (13.33 %) than in the other three blood centers (all < 3 %). The prevalence of genotype A in Urumqi was also higher than in the other blood centers (9.30 % vs. 1.79 % in Kunming, 1.67 % in Luoyang, 4.17 % in Liuzhou, and 4.44 % in Mianyang,). In Liuzhou Blood Center, no subjects with genotype D or with a genotype mixture were found.

The HBsAg subtype distribution in this study was as follows: adw (67.6 %, 95 % CI: 61.9 %-74.1 %), adr (23.3 %, 95 % CI: 17.8 %-28.7 %) and ayw (8.7 %, 95 % CI: 5.1 %-12.5 %) (Table 2). The HBV isolates from 150 of 155 (96.8 %) subtype adw subjects belonged to subtype adw2, while only four (1.8 %) belonged to adw3, and the subtype from one adw subject could not be differentiated between adw2 and adw3. All of the isolates from adr subjects belonged to subtype adrq+. The isolates from the ayw subjects were roughly evenly distributed among the subtypes ayw1, ayw2, and ayw3. The distribution of the HBV subtype varies geographically as shown in Table 1 (p < 0.0001). As with genotype, the subtype distribution in the Luoyang region is most notably different than other regions, where no statistical difference was found in the HBV subtype distribution among the other four centers (p = 0.07). In this study, eight of nine (88.9 %) genotype A isolates belonged to subtype adw2, and one to adw3. For genotype B, 123(91.8 %) viruses belonged to subtype adw2, six to ayw1, three to adw3 and one to adrq+. For genotype C, 47 (94.0 %) were adrq+, and three each belonged to subtypes adrq+ and adw2. For genotype D, six (40.0 %) belonged to ayw2, eight (53.3 %) to ayw3, and one to adw2 (Table 2). With the exception of the Luoyang Blood Center, where the proportion of adw2 and adrq+ was almost equal, subtype adw2 was predominant in all of the blood centers. The data are shown in Table 1. No subjects with subtype adw3 were found in Luoyang Blood Center, while one subject was found in each of the other four blood centers.

**Table 1:** Distribution of HBV subtypes in the five blood centers

	Subtype, N (%)								Total
	Subtotal	adw	adw3	adr	Subtotal	ayw	ayw2	ayw3	
		adw2		adrq^+^		ayw1			
Kunming	38(67.9)	37(66.1)	1(1.8)	15(26.8)	3(5.4)	2(3.6)	/	1(1.8)	56
Urumqi	33(76.7)*	31(72.1)	1(2.3)	3(7.0)	7(16.3)	2(4.7)	4(9.3)	1(2.3)	43
Luoyang	26(43.3)	26(43.3)	/	27(45.0)	7(11.7)	/	2(3.3)	5(8.3)	60
Liuzhou	19(79.2)	18(75.0)	1(4.2)	5(20.8)	/	/	/	/	24
Mianyang	39(86.7)	38(84.4)	1(2.2)	3(6.7)	3(6.7)	2(4.4)	/	1(2.2)	45
Total	155 (67.6)	150 (65.8)	4 (1.8)	53 (23.3)	20 (8.7)	6 (2.6)	6 (2.6)	8 (3.5)	228

* The subtype of one adw isolate could not be further differentiated between adw2 or adw3

**Table 2:** Relationship between HBV genotype and HBsAg subtype

Genotype	Number	Subtype, N (%)					
		adw2	adw3	adrq+	ayw1	ayw2	ayw3
A	9	8(88.9)	1(11.1)	/	/	/	/
B	134*	123(91.8)	3(2.2)	1(0.7)	6 (4.5)	/	/
C	50	3(6.0)	/	47(94.0)	/	/	/
D	15	1(6.7)	/	/	/	6(40.0)	8(53.3)
Mixture	20	15 (75.0)	/	5 (25.0)	/	/	/
Total	228	150 (65.8)	4(1.8)	53 (23.3)	6 (2.6)	6(2.6)	8(3.5)

* A sample of genotype B was determined to be subtype adw but could not be further differentiated between adw2 and adw3

### Demographic characteristics of donors associated with HBV genotypes and subtypes

**Fig. 2:**
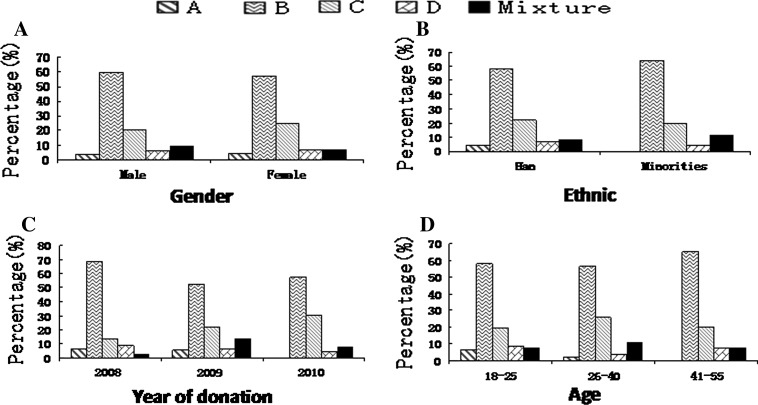
Demographic distribution of HBV genotypes. The samples were grouped by gender, ethnicity, year of donation, and age. The column represents the percent of each genotype in each subgroup

**Fig. 3:**
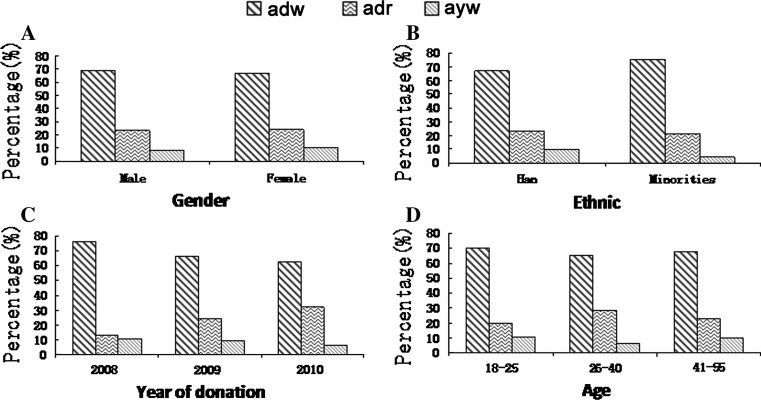
Demographic distribution of HBV subtypes. The samples were grouped by gender, ethnicity, year of donation, and age. The column represents the percent of each subtype in each subgroup

The overall distribution of HBV genotypes (p = 0.25) and subtypes (p = 0.27) were not significantly different between men and women (Figs. 2A and 3A). All nine subjects with genotype A were of the Han ethnic background; however, no statistical difference was found in the genotype distribution between Han donors and those from minority ethnic groups (Fig. 2B, p = 0.57). Figure 3B shows no statistical difference in the proportion of HBV subtypes between the Han and the minorities (p = 0.64). There was a statistically significant temporal difference in genotype distribution (Fig. 2C, p = 0.04). There appears to be a slight increase in the prevalence of subtype adr over time. There was not a statistically significant temporal difference in subtype distribution (Fig. 3C, p = 0.21). Most of the donors with genotype A were found in the 18-25 age group, and no donors infected with this genotype were found in the age group older than 40 (Fig. 2D); however, there was no statistical difference in the proportion of HBV genotypes by age (p = 0.29). Also, there was no statistical difference in the proportion of HBV subtypes by age (Fig. 3D, p = 0.52).

### Prevalence and characteristics of mutations in the S region

**Fig. 4:**
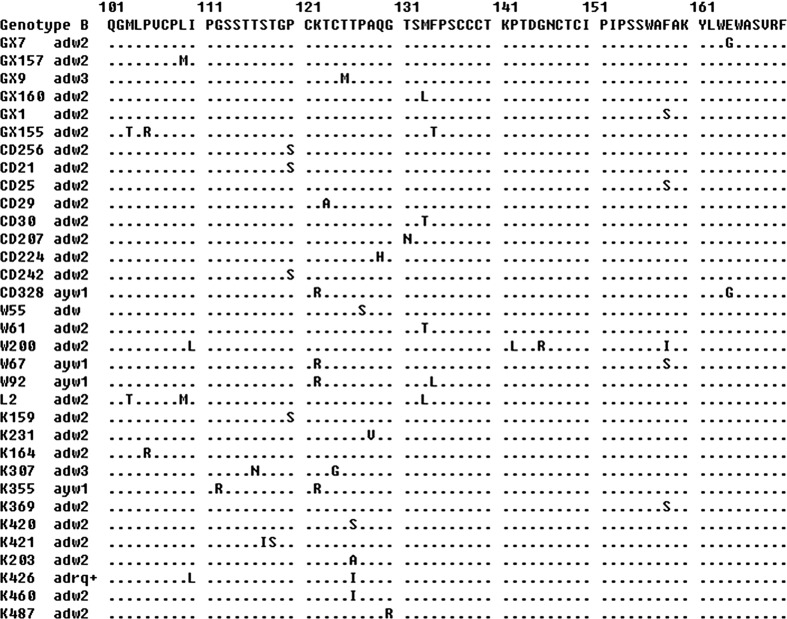
Mutations localization in the MHR of HBsAg. Urumqi, W; Kunming, K; Luoyang, L; Miangyang Blood Center, CD; and Liuzhou Blood Center, GX

Subsequent sequencing results revealed the prevalence and characteristics of the central MHR mutations among the blood donors in this study. Thirty-nine out of 228 (17.1 %) samples were found to have mutations. Fifteen (6.6 %) samples were identified with mutations that have a potential impact on the detection of HBsAg. Thirty-four of these 39 mutated strains had only one amino acid mutation in the studied region, and five had multiple mutations. There was a statistically significant difference in mutations by center (p = 0.02). The mutations were less frequent in donors at Luoyang Blood Center than at Kunming Blood Center, Liuzhou Blood Center and Mianyang Blood Center (Table 3). The mutations also varied by genotype (p = 0.02), where 31 of the viruses with MHR mutations belonged to genotype B (Table 3). The proportion of MHR mutations varied by subtype (Table 3, p = 0.001), where only 2 of 53 (3.8 %) subjects with MHR mutations belonged to subtype adrq+, and the proportion of MHR mutations was higher among subtype adw (19.4 %) and higher again among subtype ayw (35.0 %). One isolate was found with the G145R mutation, which is one of the most important mutations in the HBV S region [19]. In addition, a four-amino-acid insertion (TNRT) between amino acid position 114 and 115 was found in an isolate from the Kunming Blood Center. The alignment of amino acid sequences of the central major hydrophilic region of the 39 samples is shown in Fig. 4.

**Table 3:** Distribution of MHR mutations in the HBV S region by blood center, demographic variables, genotype, and subtype

	Number	Number of subjects with MHR mutations	Percentage (%)	p-value
Total	228	39	17.1	
Blood center				0.02
Kunming	56	13	23.2	
Urumqi	43	7	16.3	
Luoyang	60	3	5.0	
Liuzhou	24	6	25.0	
Mianyang	45	10	22.2	
Gender				0.34
Male	157	24	15.3	
Female	71	15	21.1	
Ethnicity				0.78
Han	203	34	16.7	
Minorities	25	5	20.0	
Age				0.80
18-25	107	20	18.7	
26-40	81	12	14.8	
41-55	40	7	17.5	
Genotype				0.02
A	9	1	11.1	
B	134	31	23.1	
C	50	2	4	
D	15	3	20.0	
Subtype				0.001
adrq^+^	53	2	3.8	
adw	155	30	19.4	
ayw	20	7	35.0

## Discussion

Molecular epidemiological studies provide valuable information to help understand the prevalence and characteristics of HBV genotypes and mutations. However, most of the molecular epidemiological studies of HBV in China have focused on patients, not on blood donors, who are generally asymptomatic and healthy individuals. Knowledge of the HBV genotypes and mutations in HBsAg-positive donors is important for developing effective donor screening for HBV. Studying healthy donors also provides a window into the molecular epidemiological evolution of HBV in the general population, and therefore, screening healthy blood donors should be a part of the comprehensive surveillance program. We filled this gap with a multi-center study of the geographical and demographic distribution of genotypes, subtypes and mutations in the S region of the HBV genome in donors from five blood centers, where the blood donors were geographically, socially, economically and ethnically diverse.

Although sequencing of the whole genome or S region is the gold standard for determining the HBV genotype, it is difficult to detect genotype mixtures by this method. In our study, a multiplex PCR assay was used to detect genetic mixtures in samples with heterogeneous sequences. Using this method, we detected 20 samples with genotype mixtures.

Genotypes C and B were the major HBV genotypes endemic in Mainland China. As shown in our study, HBV genotype B is more prevalent than genotype C in blood donors, while other studies have reported that genotype C has a higher prevalence in patients than genotype B. This difference may be due to the greater virulence of HBV genotype C than B [19–21]. Genotype C is associated with the development of cirrhosis and hepatocellular carcinoma as well as a lower response rate to interferon therapy. It also has a lower rate of seroconversion from HBeAg to anti-HBe and a higher HBV DNA level compared to genotype B [22]. Individuals infected with HBV genotype B may be more likely to remain asymptomatic and become part of the donor pool, posing a substantial threat to the safety of the blood supply.

Genotypes of HBV are generally subtype specific, although some subtypes are heterogeneous. In general, subtype adw is usually found in genotypes A and B, while adr occurs in genotype C [22]. In this study, we found that all of the genotype A strains and most of the genotype B HBV strains belonged to subtype adw, and most of the genotype C strains belonged to subtype adr, which is similar to previously published reports [22].

HBsAg is the main target for virus neutralization, either by natural or vaccine-induced anti-HBs. The basic working model is that of a protein with four transmembrane helices in which several residues at the N- and C-termini and a central major hydrophilic region (MHR) from residues 103-173 are exposed at the surface of viral particles. The MHR is composed of five regions: HBs1, HBs2, HBs3, HBs4 and HBs5. Mutations in this region occurring naturally or under immunization pressure could affect HBsAg test results or an individual’s reaction to vaccination [17]. In our study, about 17 % of samples were found to have mutations, and most of the mutations were located at loci that might be related to the failure of immunization and HBsAg detection. The mutation rate was lower than those reported elsewhere, including Japan [23] (24 %), Korea [24] (50 %), France [25] (28 %) and Spain (40 %) [26]. However, the lower mutation rate might also be due to differences in the length of the fragment that was analyzed. It is also possible that our mutation rate is an underestimation, because this study only included HBsAg-positive donors, while there may be HBsAg-false-negative donors who were missed by the screening due to the mutations in the “a” determinant region. In addition, the sequences were cloned, and only one clone was selected for sequencing for the samples that showed heterogeneity by direct sequencing. This approach may miss some mutations and decrease the rate of detection of mutations. The most important and best-documented mutation in MHR was G145R, which is also the most critical substitution to prevent HBsAg detection [27]. We detected this mutation in only one sample from the Urumqi Blood Center. Since G145R is associated with false negative HBsAg screening, it may be reasonable to assume that there may be infected donors with this mutation who were not included in our study because they had a false negative HBsAg screening result.

There are several potential limitations to this study. Due to low viral loads or mutations in the primer-recognition sites, a total of 17 (6.9 %) of the 245 selected HBsAg-positive samples were not genotyped successfully. Although it is possible that some of these 17 samples may be false positive for HBsAg, new primers and a more sensitive genotyping method may be useful in further studies. Because complete genome sequencing is not applicable for routine investigations, we have limited our analysis to a part of the S gene that has been described as reliable for genotyping [7]. Sequencing of a larger region will be necessary to identify subgenotypes accurately. In our study, the genotype of an isolate from Urumqi Blood Center could not be determined by sequencing because of the low bootstrap value with any genotype from A to G, but it was genotyped successfully as genotype B using multiplex PCR. However, complete genome sequencing is needed to clarify the genotype of this subject.

This study is the first multi-center study of HBV genotypes, subtypes and mutations in voluntary Chinese blood donors from representative regions throughout China. The study results were analyzed in the context of donors’ demographic and geographic characteristics. We have identified viral envelope mutants in this study that may compromise HBsAg detection in HBV-infected donors. However, HBV with envelope mutations can be detected by the nucleic acid test (NAT), which currently is not included as a test for routine blood donor screening. Our data suggest that the addition of the NAT to the routine donor screening process would minimize false negative HBV results due to S region mutations and would further increase blood safety in China. Meanwhile, large-scale molecular epidemiological studies of HBV in Chinese donors as well as in the general population will help to understand the diversity and distribution of HBV mutants and are essential to reduce the risks of transfusion-transmitted HBV.

## References

[1] Lee WM (1997). Hepatitis B virus infection. N Engl J Med.

[2] World Health Organization (2004) Hepatitis B vaccines. Wkly Epidemiol Rec 79 (28):255–263 15344666

[3] Lu J, Zhou Y, Lin X, Jiang Y, Tian R, Zhang Y, Wu J, Zhang F, Wang Y, Bi S (2009). General epidemiological parameters of viral hepatitis A, B, C, and E in six regions of China: a cross-sectional study in 2007. PLoS One.

[4] Kramvis A, Kew M, Francois G (2005). Hepatitis B virus genotypes. Vaccine.

[5] Yim HJ (2008). Hepatitis B virus genetic diversity and mutant. Korean J Hepatol.

[6] Norder H, Courouce AM, Coursaget P, Echevarria JM, Lee SD, Mushahwar IK, Robertson BH, Locarnini S, Magnius LO (2004). Genetic diversity of hepatitis B virus strains derived worldwide: genotypes, subgenotypes, and HBsAg subtypes. Intervirology.

[7] Lindh M, Andersson AS, Gusdal A (1997). Genotypes, nt 1858 variants, and geographic origin of hepatitis B virus–large-scale analysis using a new genotyping method. J Infect Dis.

[8] Yu H, Yuan Q, Ge SX, Wang HY, Zhang YL, Chen QR, Zhang J, Chen PJ, Xia NS (2010). Molecular and phylogenetic analyses suggest an additional hepatitis B virus genotype “I”. PLoS One.

[9] Tatematsu K, Tanaka Y, Kurbanov F, Sugauchi F, Mano S, Maeshiro T, Nakayoshi T, Wakuta M, Miyakawa Y, Mizokami M (2009). A genetic variant of hepatitis B virus divergent from known human and ape genotypes isolated from a Japanese patient and provisionally assigned to new genotype J. J Virol.

[10] Zeng G, Wang Z, Wen S, Jiang J, Wang L, Cheng J, Tan D, Xiao F, Ma S, Li W, Luo K, Naoumov NV, Hou J (2005). Geographic distribution, virologic and clinical characteristics of hepatitis B virus genotypes in China. J Viral Hepat.

[11] Liang X, Bi S, Yang W, Wang L, Cui G, Cui F, Zhang Y, Liu J, Gong X, Chen Y, Wang F, Zheng H, Guo J, Jia Z, Ma J, Wang H, Luo H, Li L, Jin S, Hadler SC, Wang Y (2009). Evaluation of the impact of hepatitis B vaccination among children born during 1992–2005 in China. J Infect Dis.

[12] Guo N, Wang J, Ness P, Yao F, Dong X, Bi X, Mei H, Li J, He W, Lu Y, Ma H, Wen X, Huang M, Wright DJ, King M, High P, Nelson K, Shan H (2011). Analysis of Chinese donors’ return behavior. Transfusion.

[13] Thompson JD, Gibson TJ, Plewniak F, Jeanmougin F, Higgins DG (1997). The CLUSTAL_X windows interface: flexible strategies for multiple sequence alignment aided by quality analysis tools. Nucleic Acids Res.

[14] Tamura K, Dudley J, Nei M, Kumar S (2007). MEGA4: molecular evolutionary genetics analysis (MEGA) software version 4.0. Mol Biol Evol.

[15] Chen J, Yin J, Tan X, Zhang H, Chen B, Chang W, Schaefer S, Cao G (2007). Improved multiplex-PCR to identify hepatitis B virus genotypes A-F and subgenotypes B1, B2, C1 and C2. J Clin Virol.

[16] Norder H, Hammas B, Lofdahl S, Courouce AM, Magnius LO (1992). Comparison of the amino acid sequences of nine different serotypes of hepatitis B surface antigen and genomic classification of the corresponding hepatitis B virus strains. J Gen Virol.

[17] Echevarria JM, Avellon A (2006). Hepatitis B virus genetic diversity. J Med Virol.

[18] Kay A, Zoulim F (2007). Hepatitis B virus genetic variability and evolution. Virus Res.

[19] Mayerat C, Mantegani A, Frei PC (1999). Does hepatitis B virus (HBV) genotype influence the clinical outcome of HBV infection?. J Viral Hepat.

[20] Locarnini SA (2002). Clinical relevance of viral dynamics and genotypes in hepatitis B virus. J Gastroenterol Hepatol.

[21] Kobayashi M, Arase Y, Ikeda K, Tsubota A, Suzuki Y, Saitoh S, Suzuki F, Akuta N, Someya T, Matsuda M, Sato J, Kumada H (2002). Clinical characteristics of patients infected with hepatitis B virus genotypes A, B, and C. J Gastroenterol.

[22] Kao JH, Chen PJ, Lai MY, Chen DS (2000). Hepatitis B genotypes correlate with clinical outcomes in patients with chronic hepatitis B. Gastroenterology.

[23] Ogura Y, Kurosaki M, Asahina Y, Enomoto N, Marumo F, Sato C (1999). Prevalence and significance of naturally occurring mutations in the surface and polymerase genes of hepatitis B virus. J Infect Dis.

[24] Song BC, Kim SH, Kim H, Ying YH, Kim HJ, Kim YJ, Yoon JH, Lee HS, Cha CY, Kook YH, Kim BJ (2005). Prevalence of naturally occurring surface antigen variants of hepatitis B virus in Korean patients infected chronically. J Med Virol.

[25] Roque-Afonso AM, Ferey MP, Ly TD, Graube A, Costa-Faria L, Samuel D, Dussaix E (2007). Viral and clinical factors associated with surface gene variants among hepatitis B virus carriers. Antivir Ther.

[26] Avellon A, Echevarria JM (2006). Frequency of hepatitis B virus ‘a’ determinant variants in unselected Spanish chronic carriers. J Med Virol.

[27] Ly TD, Servant-Delmas A, Bagot S, Gonzalo S, Ferey MP, Ebel A, Dussaix E, Laperche S, Roque-Afonso AM (2006). Sensitivities of four new commercial hepatitis B virus surface antigen (HBsAg) assays in detection of HBsAg mutant forms. J Clin Microbiol.

